# Pulp Mummification

**Published:** 1900-01-15

**Authors:** Theodore Soderberg

**Affiliations:** Sydney, Australia


### PULP MUMMIFICATION.

BY THEODORE SODERBERG, SYDNEY, AUSTRALIA.

In the *Dental Cosmos* for May, 1893, Dr. W. E. Christensen communicated some comments on the Witzel and Herbst methods of treating devitalized pulps, and pointed out that the real effect aimed at by the two German dentists was the *mummification* of the pulps left untouched in root canals.

Previous to reading about "this treatment, which we may call simple, and at the same time scientific," I had followed the usually thought and practiced method of treating devitalized pulps, viz., to give my patients just the amount of strain and pain equivalent to the amount of my own bull-dog perseverance with that exquisite instrument of torture—the nerve-broach; then *trying* to fill the canals with gutta-percha or asbestos fibers saturated with some antiseptic; and then—well, the final result was not always my patient's comfort and my own glory.

I discarded the old method and adopted Dr. Witzel's, commencing by using the following modified formula (his paste, as formulated by Dr. Christensen, being too thin to be workable):

R. Hydrarg. bichlor. corr., gr. xxx;
Morph. muriat., . . . gr. xv;
Ac. phenyl., . . . . . gr. x;
Ol. menth. pip., . . . gtt. j;
Ol. caryophyl., . . . gtt. j;
Alum, exsiccat., q. s. to make stiff paste.

This paste had, however, the drawback which all pastes containing mercury have—it caused discoloration of the tooth, at least when used in connection with steel instruments and amalgam.

Shortly afterward (September, 1893), the *Dental Cosmos* brought out Dr. W. D. Miller's valuable contribution, "Concerning Various Methods Advocated for Obviating the Necessity of Extracting Devitalized Tooth-pulps," and I at once commenced to experiment with different pastes to find one which would cause mummification of the pulp without discoloration of the tooth.

Besides Dr. Witzel's paste, as given above, I have experimented with three of Dr. Miller's formulæ, using glycerol as the binding agent, viz:

| | | |
|---|---|---|
| Sublimate, } equal parts; | Sublimate, } | Ol. cassia, |
| Thymol, } | Thymol, } equal parts; | Thym'l, q. s. |
| Glycerol, q. s. | Tannin, } | |
| | Glycerol, q. s. | |

Here, again, the disadvantage is, as pointed out by Dr. Miller, the discoloration of the tooth—bluish black by the mercury, yellowish-brown by the oil of cassia. My test-tubes further show that where tannin is used in connection with either of these, or any other paste—especially if glycerol be used as the binding agent—the discoloration is more marked.

Experiments with twelve other pastes, first in test-tubes, next with freshly-extracted teeth, proved to my preliminary satisfaction that the following formula met all conditions most acceptably:

℞ Dried alum, . . . ʒi;

Thymol, . . . . . ʒi;

Glycerol, . . . . . ʒi;

Zinc oxid, q. s. to make stiff paste.

In this paste the thymol acts as the antiseptic, the alum as the mummifying agent, the zinc oxide as the coloring medium, and the glycerol as the binding and penetrating agent.

Dr. Bennette, of Berkenhead, England, recommends mixing a small quantity of paraform, which is polymerized formaldehyde, with Soderberg's paste.

Bearing in mind Dr. Miller's favorable recommendation of thymol, I adopted it as my antiseptic, and I fancy that I shall not have cause to regret my choice. My reason for adopting dried alum was that its tanning properties are far superior to those of tannin or any other tanning agent—a statement which any practical tanner will indorse. Further, dried alum does not discolor the paste, while tannic acid, if substituted for the alum in the above mixture, produces a dark-brown paste.

I need hardly point out that glycerol, with its great affinity for moisture and its well-known penetrative power, is an excellent carrying agent for all mummification pastes; further, that no better coloring medium can be found for the purpose in view than zinc oxide.

The entire paste is, finally, non-irritating; I have, in fact, continually used it for other purposes, *e. g.*, in deep cavities between pulp and filling material, etc.

I have used this particular paste for over twelve months, and, *so far,* not one single case out of a total of ninety-seven has given any after-trouble (alveolar abscess). And here I may be allowed to state that neither have I, so far, had any after-trouble with any of the cases treated with Dr. Witzel's paste (from August, 1893, to date). I am perfectly well aware that neither one year, nor two, is sufficient time to warrant a guarantee of absolute success, and I acknowledge the truth of Dr. Miller's words, "Granted that the pulp becomes sterilized by the application, this does not say that it remains sterile indefinitely."

My patients mostly belong to the so-called middle and working classes. For divers reasons, they are not as careful with their teeth as are the members of the more cultured and moneyed classes; they don't, as a rule, think of visiting the dentist except when an exposed pulp acts as a vivid reminder. In short, my practice is such a one that I have to resort to my devitalizing paste very frequently. Hence excellent opportunities to use and observe the effect of the mummification paste. (And hence, also, the "kind o' sickly smile" wherewith I point out to my pupils Dr. Miller's angelic advice: "One or two cases every month—at least for the first year or two—is all that a careful dentist ought to risk in private practice." It is, however, a great consolation to me to read in the same article that over two hundred cases were treated with Dr. Miller's paste at the Dental Institute of the University of Berlin, with only one failure on record). I have tested the action of the mummification paste in the way that conforms best with my bent of mind and my inferior standard of scientific education; I have removed test fillings three, six, nine and twelve months after the pulp-treatment took place. In all cases the same satisfactory result observed—mummification of the pulp. One case I especially wish to record. I had occasion to extract an upper third molar treated seventeen days previously. On immediately splitting the tooth, I found the pulp in the root-canals perfectly mummified down to the very foramina. Whether the experiments are carried out with teeth *in situ*, or with extracted teeth, the mummified pulps always present the same appearance, viz., a perfectly dry, parchment-like mass, with a faint odor of thymol, and a whitish color.

My mode of procedure is as fellows: After the pulp has been perfectly devitalized (arsenic, cocain, alum, equal parts, glycerol, q. s., sealing with sticky wax), I open up the main pulp-chamber and drill out its dead contents, leaving the root-canals untouched. I then fill the chamber with paste, and with a flexible Donaldson bristle gently prick the paste into the pulps left in the canals (this, however, not absolutely necessary). I now seal with cement and insert amalgam or gold, as the case may be. I use rubber-dam or cup where possible, otherwise Sperling's rolls—the main thing being to keep out the saliva, at least until the first piece of amalgam has been inserted and burnished round.

Again I must turn to Dr. Miller's article for a valuable quotation: "No dentist who has an appreciation of the needs of the public at large, and does not confine his sphere of thought to the limits of his own practice (with fifty to one hundred dollars for treating a tooth), can question the statement that our present methods of treating many molars with dead pulps are difficult, expensive and, in the hands of the majority, uncertain. The only alternative of extirpating the pulp is leaving it in. Having left the pulp in the canal, shall we leave it in the shape of a putrescible matter, or shall we endeavor, in some way, to render it non-putrescible? The latter method appears to me to be the more rational." If this is not granite logic, I, for one, am at a loss whither to turn to find it.

The following extracts from an article published in the *Items of Interest* for October, 1898, page 742, by Dr. J. A. Waas are valuable as evidence from the manipulative point of view.

The subject of pulp mummification has not, so far at least as I have been able to ascertain, been taken up to any great extent by our profession in the United States. A brief account of my experience with this method of treatment during the past three years may be of interest to some.

Among the members of the profession who first introduced and advocated the treatment were Drs. Herbst, Miller and Soderberg, and I consider that they have laid the dental profession of the present day under a debt of gratitude for their researches and experiments which have resulted in bringing the practice of pulp mummification to its present advanced stage.

I myself, after three years' practice of the treatment, am more enthusiastic than ever in its praises, not only because it lessens the pain and suffering on the part of the patient, but because of the satisfaction I have in knowing that when a tooth is once treated in this way it is, I think, so far as regards the pulp structure of the tooth, treated for all time, the pulp being as harmless and inert as the subjects of King Pharoah shut up in the Pyramids.

The treatment is of especial value in cases of obscure, supplementary or unsuspected branches of the pulp, which, under the old removal method, are simply beyond the reach of the highest dental skill, but which, under the mummification treatment are rendered harmless even though their presence is unknown to and unsuspected by the operator.

The treatment I am using is that recommended by Dr. Soderberg, of Sydney, Australia.

I follow the directions of Dr. Soderberg to the letter, and of sixty-one cases which I have treated during the past three years to April 1, 1898, I have not met with *one failure* so far, not even *one* complaint of tenderness. Such a positive statement as this I can not make regarding my previous work of filling pulp canals, although I think that I have always been as careful as the average operator.

The patients treated by me ranged in age from six years to fifty-five or sixty years, and were in every condition of health and illness, from that of robust vigor to a late stage of tuberculosis.

Dr. Waas here schedules some sixty cases, giving characteristics of patients, operations and results. This schedule can be consulted in the *Items of Interest* as noted above.

The results achieved have been ample warrant for my favorable view of pulp mummification. I can not say what the future may develop, but this is a remarkable record, to treat so many teeth without one of the patients having had even a moment of tenderness on account of a tooth so treated from the time of leaving my office to the present.

The greater number of these cases have, of course, been patients in my own town, and some of them have been in my office several times since the treatment for other operations, and I was always anxious to examine the teeth and inquire as to their condition, and have certainly felt more than gratified to hear nothing but favorable reports.

The successful treatment of dead pulps by mummification is not the only benefit derived from this method of treatment, for besides its effectiveness, it is a great saver of both time and labor, and as all of us are not blessed with a practice which permits us to charge what we please, I consider this an important factor in enabling us to bring our charges down to a reasonable basis and within the means of those from whom the bulk of our practice is derived.

As to the saving of time it seems manifest that this must follow from a method which permits the treatment and filling of the tooth in half the time or less than would be required to remove the pulp and fill the canal, while the wear and tear upon the nerves of the operator himself are reduced to a minimum, because of the serene satisfaction that he feels in knowing that what has hitherto been the most painful and difficult part of the operation is now being done without causing either the patient pain or the operator inconvenience.

The assertion has been made time and again by old practitioners that apical ends of the pulps must of necessity remain in teeth when they cannot be removed, and it is also true that broaches, root reamers, and other dental instruments, when broken off, have been left in canals for years without causing any trouble. If such is really the case (and we all know that it is, although we very reluctantly admit it) why should we not try to permit the pulp itself to remain, in a hardened, dried-up condition? The process of mummification is surely worth a trial and I feel certain that any operator who will look the matter up in the article referred to will feel amply repaid for the trouble and will be thankful to resort at some time to the mummification of a pulp that is impossible to remove from some inaccessible cavity, instead of being compelled to extract the tooth in order to give relief.

The following paper by Prof. Boennecken is reprinted entire from the *Dental Cosmos* of May, 1899, because it gives the recent view of the matter from the German point of view:

